# Diet App Use by Sports Dietitians: A Survey in Five Countries

**DOI:** 10.2196/mhealth.3345

**Published:** 2015-01-22

**Authors:** Michelle R Jospe, Kirsty A Fairbairn, Peter Green, Tracy L Perry

**Affiliations:** ^1^Department of Human NutritionUniversity of OtagoDunedinNew Zealand; ^2^Highlanders Super Rugby FranchiseDunedinNew Zealand; ^3^Landcare ResearchDunedinNew Zealand; ^4^Department of Mathematics and StatisticsUniversity of OtagoDunedinNew Zealand

**Keywords:** nutritional requirements, nutrition assessment, dietary self-monitoring, mobile apps, questionnaire, telemedicine, sports nutritional sciences

## Abstract

**Background:**

Despite the hundreds of diet apps available for use on smartphones (mobile phones), no studies have examined their use as tools for dietary assessment and tracking in sports nutrition.

**Objective:**

The aim is to examine the prevalence and perceptions of using smartphone diet apps for dietary assessment and tracking among sports dietitians.

**Methods:**

A cross-sectional online survey to examine the use and perception of diet apps was developed and distributed to sports dietitians in Australia, Canada, New Zealand, the United Kingdom, and the United States (US).

**Results:**

The overall response rate from the 1709 sports dietitians invited to participate was 10.3% (n=180). diet apps were used by 32.4% (57/176) of sports dietitians to assess and track the dietary intake of athletes. Sports dietitians from the US were more likely to use smartphone diet apps than sports dietitians from other countries (OR=5.61, 95% CI 1.84-17.08, *P*=.002). Sports dietitians used 28 different diet apps, with 56% (32/57) choosing MyFitnessPal. Overall, sports dietitians held a positive perception of smartphone diet apps, with the majority of respondents viewing diet apps as “better” (25/53, 47%) or “equivalent” (22/53, 41%) when compared with traditional dietary assessment methods.

**Conclusions:**

Nearly one-third of sports dietitians used mobile phone diet apps in sports nutrition practice, and viewed them as useful in helping to assess and track the dietary intake of athletes.

##  Introduction

Dietitians are experts in nutrition and work in all sectors of health [1]. Sports dietitians advise athletes on appropriate and effective nutrition for health, physical activity, and athletic performance [2]. Sports nutrition is prescriptive, with nutrition recommendations set as grams of macronutrients (such as protein, carbohydrates, and fat) per day based on an athlete’s characteristics [2]. This makes sports nutrition a field that benefits from the quantitative assessment of energy and nutrient intakes.

A core practice of sports dietitians is to assess dietary intake by examining the composition and adequacy of food and nutrients usually consumed by an athlete. However, accurate dietary assessment using traditional methods (such as a diet record recorded with pen and paper) is challenging as people tend to misreport the type and amount of food consumed, either because of memory lapse, social desirability bias, incorrect estimation of portion size, or lack of knowledge of the content of meals. Additionally, people may alter their usual food and fluid intake to ease recording [3-5]. Furthermore, traditional methods require dietitians to convert food intake to nutrient intake, which can be labour-intensive and prone to error [6].

Sports dietitians also ask athletes to track or monitor their dietary intake after implementing nutrition advice, in order to evaluate the effect and adherence to prescribed dietary recommendations [7]. Interestingly, an additional benefit of tracking dietary intake is that this behavior may increase an athlete’s adherence to nutrition recommendations, as dietary self-monitoring has been shown to result in greater achievement of nutrition and body composition goals [8-11].

The emergence of smartphone (mobile phone) technology and diet apps has expanded the tools available to sports dietitians to assess and track dietary intake. Diet apps on smartphones allow recording of food intake, which is instantly converted to nutrient intake and compared with calculated nutrition goals. Nutrition goals are calculated based on a diet app user’s sex, weight, weight goals, and activity level. Food entries and weight progress can be shared with a dietitian in real time [12].

Studies examining the validity of diet apps on personal digital assistants (PDAs) have shown them to be as valid as traditional dietary assessment methods [13,14], however whether this also applies to use on smartphones is not well established. To date, only one study has examined the validity of a data-based smartphone diet app, and found that the diet app correlated highly with 24-hour recalls (a traditional dietary assessment method) for estimating group means of energy and macronutrient intakes, but showed wide limits of agreement with individual energy intakes [15]. These results suggest that the specific diet app tested may not be valid for assessing individual dietary intake, however whether this apply to other diet apps is untested.

Regardless of their uncertain validity, smartphone diet apps have become prolific in app stores [12] and popular among people seeking dietetic advice [16]. Despite their popularity, no studies have examined their use as tools for dietary assessing and tracking in sports nutrition. Therefore, we conducted an international survey with the aim of examining prevalence and perception of using smartphone diet apps for dietary assessment and tracking among sports dietitians.

## Methods

### Overview

A cross-sectional online survey to assess smartphone diet app use in sports dietetics was developed and distributed electronically between June 22 and November 11, 2012, to sports dietitians in Australia, Canada, New Zealand, the United Kingdom (UK), and the United States (US). We selected English-speaking countries that had known sports dietetic associations. An administrator from each dietetic association sent a series of emails to their members to invite them to participate in our survey. We chose to survey registered sports dietitians, as their registration guarantees academic and professional qualification in nutrition [1,17]. Ethical approval was obtained from the University of Otago Ethics Committee.

### Survey Design and Administration

Survey questions were initially developed by reviewing the literature on technological advancements in dietary assessment and tracking, and by pilot interviews of two registered sports dietitians, conducted in March 2012. Semi-structured interviews lasted one hour each and included the following topics: methods of dietary assessment and tracking in sports dietetics, barriers in assessing and tracking dietary intake of athletes, and benefits and limitations of using smartphone diet apps. The interviews were recorded (with Philips Voice Tracer digital recorder lfh0622), transcribed, and analyzed.

The resulting questionnaire (Multimedia Appendix 1) asked sports dietitians about their use of smartphone diet apps in assessing and tracking dietary intake of athletes, dietary assessment and nutrition intervention in sports dietetics, and demographic information. The questionnaire was refined based on recommendations from survey design best practices [18-21], and from pretesting with a convenience sample of seven people, consisting of a sports dietitian, a clinical dietitian, a sports nutritionist, a clinical psychologist, a biostatistician, and two second-year Master of Dietetics students. The purpose of pretesting was to determine whether the questionnaire was clear, concise, and user-friendly. Amendments included: changing “tick all that apply” questions to forced-choice, dichotomous questions (eg, yes or no); reordering questions to ensure that the most important questions were asked at the start of the survey; adjusting wording to increase comprehension; and adding multiple-choice answers to cover a wider range of possible responses.

Skip logic, which is also known as “adaptive questioning”, was employed to direct respondents through different paths in the questionnaire based on their answers. The questionnaire included a total of 27 questions. Depending on the respondent’s answers, the number of questions presented ranged from 13 to 26. Of the questions, 9 required an answer before the respondent could proceed to the next page. The questionnaire was 11 pages, with 1 to 8 questions per page. Question order was not randomized because of the use of skip logic. However, answer choices for multiple-choice questions were randomized or flipped [19]. The questionnaire was set to only allow one response per computer using secure sockets layer data encryption, which limited responses based on Internet protocol addresses. Respondents were able to review their answers prior to submitting the questionnaire with the use of a “previous” button. Respondents were permitted as much time as they required to complete the questionnaire, but they could not re-enter the questionnaire after it was exited.

The questionnaire took 5-15 minutes to complete, depending on the paths taken through the questionnaire. A progress bar appeared on every page, which displayed the percentage of the questionnaire that was complete. A ‘thank you’ page was automatically displayed upon completion of the questionnaire. A separate collector was set up for each dietetic association that consisted of customized weblinks, identifying the dietetic membership of respondents. Administrators of each dietetic association sent their members an invitation email with a link to the questionnaire. Reminder emails were sent to all respondents one and two weeks after the initial email was distributed, following the Dillman’s Tailored Design Method [19]. Respondents had four weeks to complete the questionnaire.

Respondents who completed the questionnaire could submit their email addresses to receive a factsheet about using smartphone diet apps in sports dietetics practice, a background paper on dietary tracking with smartphones, and results from the study. Email addresses were collected with a second questionnaire, which was hyperlinked to the end of the primary questionnaire (*“click here to enter your email address”*). The primary questionnaire remained anonymous, as it was not associated with the second questionnaire.

### Statistical Analysis

Results from both complete and incomplete questionnaires were included in the final analysis. Response rate was calculated as the number of first question respondents, divided by the number of invited respondents. Completion rate was calculated as the number of last page respondents, divided by the number of first page respondents. Data was analyzed using Stata version 12 (StataCorp LP, College Station, TX, USA). Descriptive statistics were used to describe attitudes and current practices of sports dietitians (including frequency counts and cross-tabulations). Associations were examined using Fisher’s exact test, as cell counts were frequently less than five. All statistical comparisons were 2-tailed, and *P*<.05 was considered statistically significant. No weighting scheme was used. Text responses were analyzed qualitatively and coded. Responses to open-ended questions about the benefits and limitations of diet apps were clustered into common themes and paired with representative quotes from respondents.

## Results

### Response

The overall response rate from the 1709 sports dietitians invited to participate was 10.3% (n=180). Of the 180 respondents who began the questionnaire, 90.0% (n=162) completed it. Four respondents were excluded from analysis because they were dietetic students and were not yet working. The results presented include both complete and incomplete questionnaires, yielding 176 responses.

### Respondents

The plurality of respondents were Australian or Canadian, in the age range of 30-39 years, and had been practicing sports nutrition for 6 to 10 years (Table 1). Responding sports dietitians most frequently advised clients performing endurance type sports: running (63/158, 39.9%), triathlon or multisport (53/158, 33.5%), and cycling (31/158, 19.6%).

**Table 1:** Demographics of survey respondents.

Characteristic (N)^a^		N	%
**Country (N=174)**			
	Australia	54	30.7
	Canada	54	30.7
	United States	39	22.2
	United Kingdom	17	9.7
	New Zealand	10	5.7
**Age (N=162)**			
	21-29	40	24.7
	30-39	55	34.0
	40-49	36	22.2
	50-59	22	13.6
	60 or older	9	5.6
**Years Practicing (N=161)**			
	0-1	14	8.7
	1-5	22	13.7
	6-10	54	33.5
	11-15	36	22.4
	16-20	16	9.9
	More than 20	16	9.9
Other (part-time)		3	1.9

^a^ Results reflect the proportion of respondents who answered each question.

### Smartphone Diet App Usage

For this section all results refer to the subset of participating sports dietitians who use diet apps in their clinical practice (n=57) and are referred to as *diet app users.*

**Figure 1:**
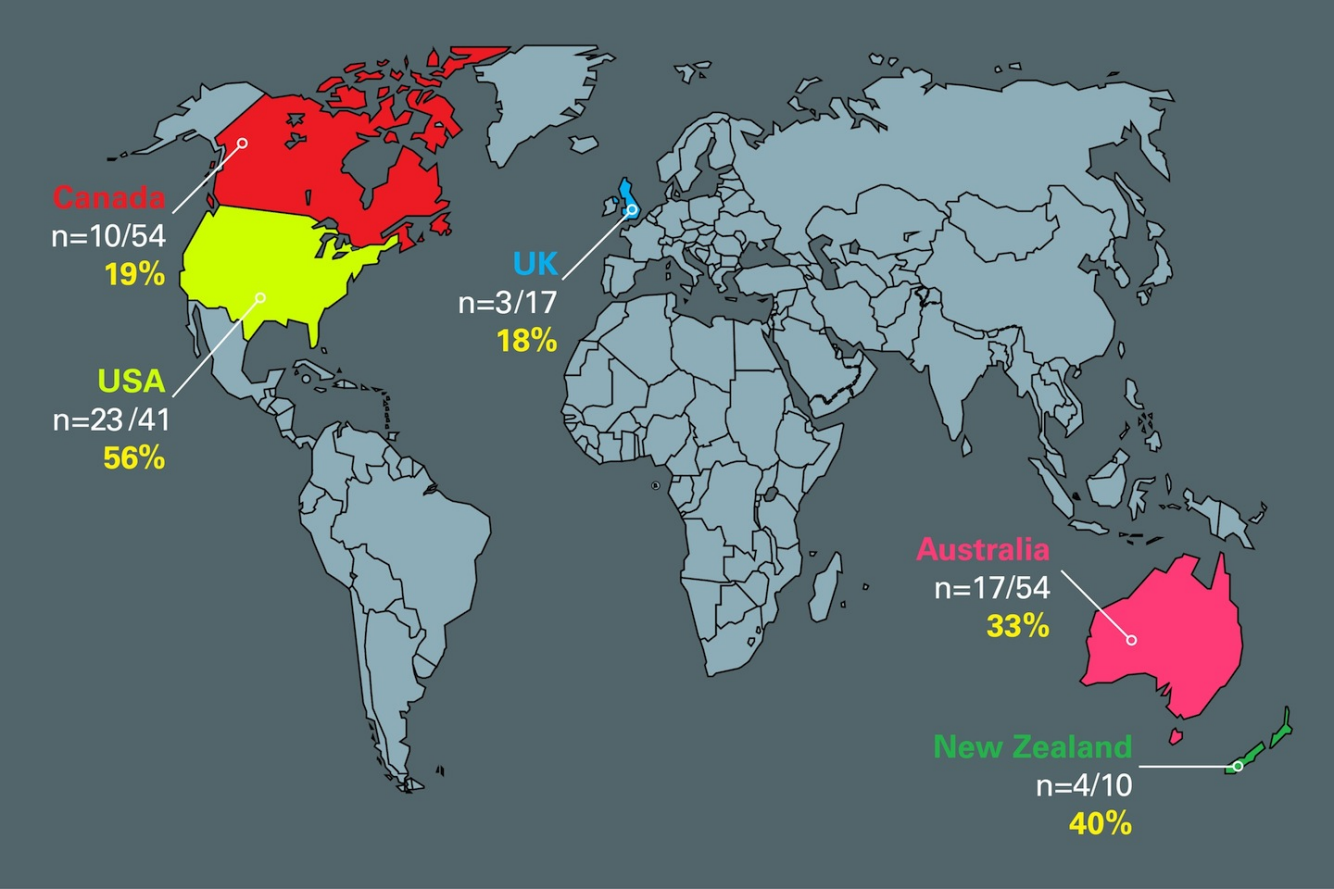
Diet app use by sports dietitian’s country of residence.

Smartphone diet apps were used by 32.4% (57/176) of sports dietitians to help them assess and track the dietary intake of athletes. The age of the sports dietitian was not associated with diet app use (logistic regression *P*>.05). The country of residence of the sports dietitian was significantly associated with diet app use, with dietitians from the US more likely to use diet apps (odds ratio=5.61, 95% CI 1.84-17.08, *P*=.002). This translated to 56% of US sports dietitians using diet apps versus 25% for the other countries combined (Figure 1).

**Figure 2:**
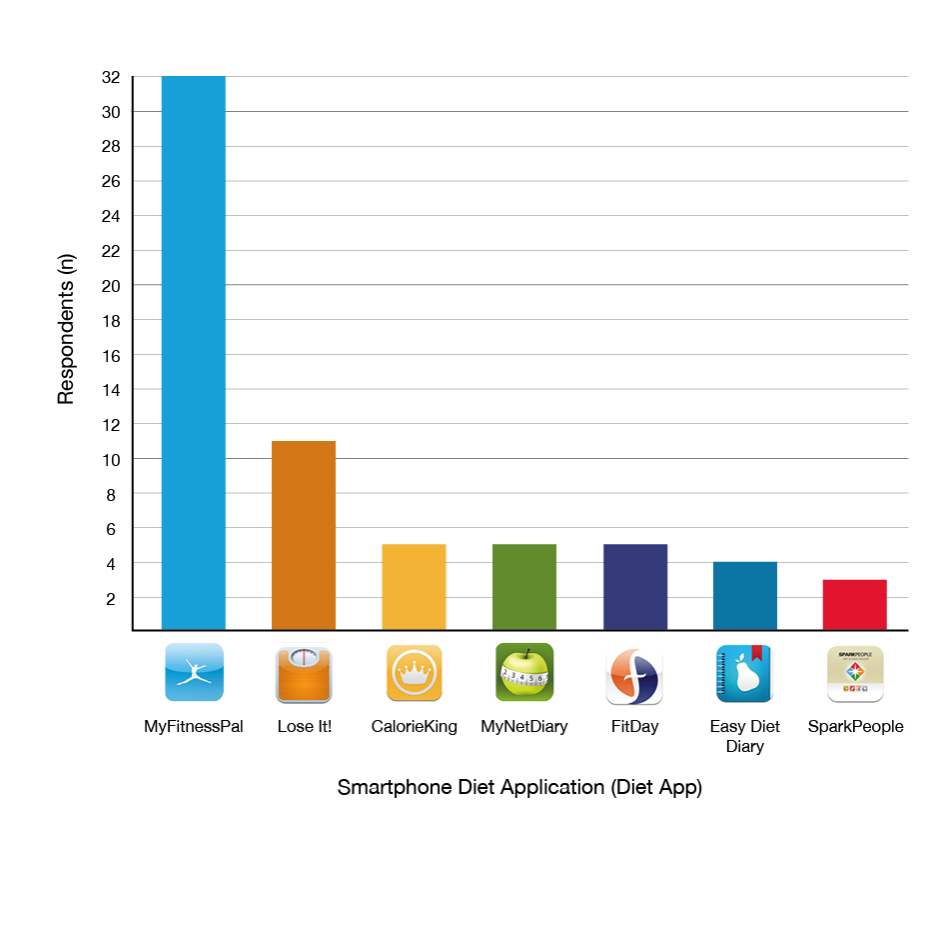
“Which smartphone diet application(s) do you use?” N=57.

Overall, diet app users utilized 28 different smartphone diet apps when counseling clients. MyFitnessPal was overwhelmingly the most popular diet app, used by 56% (32/57) of diet app users (Figure 2). In the previous three months 19/53 (36%) diet app users had recommended diet apps to 5 or less clients, 12/53 (23%) had recommended diet apps to 6-10 clients, 7/53 (13%) had recommended diet apps to 11-15 clients, 3/53 (6%) had recommended diet apps to 15-20 clients, and 12/53 (23%) had recommended diet apps to 20 or more clients.

### Perception of Diet Apps

**Figure 3:**
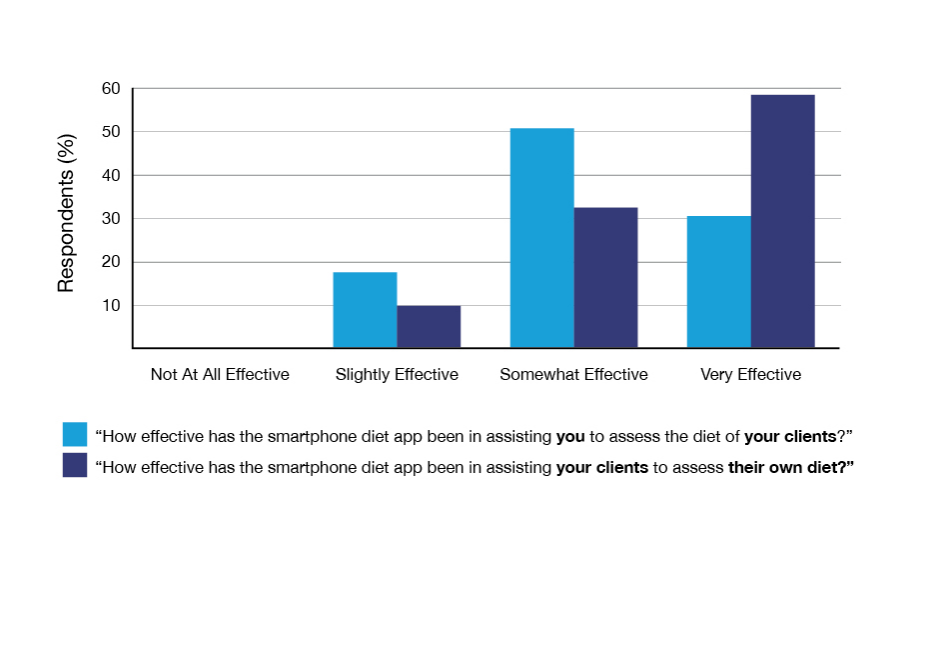
Perceived effectiveness of smartphone diet apps in assessing dietary intake (N=53).

Respondents rated the effectiveness of diet apps in assisting with dietary assessment (Figure 3). Sports dietitians rated diet apps as “very effective” for their clients assessing their own diet more often than for themselves (as sports dietitians) assessing the diet of their clients. No respondents stated that diet apps were “not at all effective”. The majority of diet app users viewed diet apps as “better” (25/53, 47%) or “equivalent” (22/53, 42%) compared with traditional dietary assessment methods, such as a diet record or diet history recorded on paper. Only 6/53 (11%) of app users rated the diet app “worse” compared with traditional dietary assessment methods.

Of the diet app users, 95% (54/57) reported limitations and benefits of using diet apps in clinical practice. The most commonly perceived limitations of diet apps were problems with the nutrient database, incorrect portion size selection by the client, and incorrect food selection by the client (Table 2). The most commonly perceived benefits of diet apps were their ubiquity, convenience, and ease of use (Table 3).

**Table 2:** “What are the limitations of using smartphone diet apps?” (N=54)^a^.

Theme	n	%	Example of Comments
Nutrient database is inaccurate, missing foods, not country-specific	22	41	“Poor, incomplete databases with no monitoring of foods that are added by users for accuracy” “Food products are not on database (but can be added)” “Some don’t contain many sports foods” “Many overseas versions and not always using foods common in New Zealand” “Lack of Canadian foods and restaurants [in the nutrient database]”
Incorrect portion size selection	12	22	“Inaccuracies in reporting portions of foods” “The patient must know portions”
Incorrect food selection	10	19	“The clients must know the differences between foods and how they were prepared” “Inaccuracies in reporting types of foods”
Client does not own smartphone	7	13	“Not all clients have smartphones” “Older or less affluent clients lack access or skills with technology”
Requires client to be tech savvy	7	13	“Difficult to use for those that are not tech savvy” “I have clients that are not as familiar with using applications” “Requires skills to use apps”
Difficulty transferring data from app to dietitian’s reports	7	13	“Getting the information off the app onto a record system that the dietitian can keep” “Getting access to the information from a client’s phone” “Transferring the results”

^a^Questions were open-ended.

**Table 3:** “What are the benefits of using smartphone diet apps?” (N=54)^b^.

Theme	n	%	Example of comments
Ubiquitous	27	50	“Most people have a smartphone and use it constantly” “Clients ALWAYS have their phone with them anyways” “Easy access for client to record, so decreases forgetting”
Convenient	14	26	“More convenient than pen and paper record” “It is more convenient so I find my clients are more likely to consistently track” “Convenient for client to enter diet records and for me to see them”
Easy to use	12	22	“They are extremely fast and easy to use encouraging adherence” “Ease of recording for athlete”
Enter foods as they are eaten	11	20	“Clients can record food as they go. Less risk of forgetting” “Clients can use it throughout their day, less likely to forget foods they have eaten”
Instant feedback	10	19	“Immediate feedback” “Instant results” “Provides feedback to the client quickly”
Increases awareness of nutrition	8	15	“Creates awareness of calories in and out” “Effects of overconsumption of treats seen very dramatically, leading to moderation” “An education tool” “Increased awareness of food composition for clients”
Accurate	7	13	“Possibly more accurate as they can record as they go and not embarrassing to be seen to be writing down food intake as everyone is on phones these days” “People can enter items in real time, which I believe improves accuracy of the diet record” “Likely more honest about the foods they have eaten”

^b^Questions were open-ended.

##  Discussion

### Principal Results

The present study is, to our knowledge, the first to survey sports dietitians about their use and perceptions of smartphone diet apps in sports nutrition practice. We found that one in three sports dietitians used smartphone diet apps to assess and track the dietary intake of athletes. This prevalence is comparable to a recent study by Lieffers et al [22] that surveyed Canadian dietitians from all disciplines and found that 40.5% of respondents had recommended diet apps to clients.

In our study, MyFitnessPal was the most popular diet app, which is consistent with finding from Lieffers et al [22]. As far as we know, there are no commercially available apps designed specifically for sports nutrition. Sports dietitians and athletes are required to use general diet apps instead, which are often designed for weight loss [23]. This presents an interesting gap in the app market as sports nutrition has particular requirements that could be highlighted in an app, specifically: the timing of food intake (especially before, during, and after exercise); the use of sports foods/drinks and ergogenic supplements (eg, tracking the amount of caffeine consumed); energy balance, including signs of low energy availability (eg, loss of menstrual function); and the ability to easily manipulate nutrient goals based on training and competition (eg, carbohydrate loading, rehydration, recovery nutrition, etc) [7].

Sports dietitians in our survey believed that the ubiquity of diet apps may increase the accuracy of dietary assessment/tracking. The immediate entry of records after eating would reduce reliance on memory, which is notoriously problematic in traditional dietary assessment methods [5]. While using PDAs to record meals does not eliminate erroneous food entry [24], further research is needed on diet apps to gauge accuracy and immediacy of diet recording after meals. Concern about accuracy of diet apps was evident in our survey, where accuracy of the nutrient database and incorrect food entry by athletes were the main reported limitations of diet apps, and is in agreement results from Lieffers et al [22]. This highlights the need for further studies to validate commercially available diet apps for dietary assessment and tracking, particularly among athletes.

In the present survey, sports dietitians had a positive perception of diet apps in helping them to assess/track dietary intake, which was in agreement with Lieffers et al [22] who similarly found that reported benefits included convenience and ease of use. If diet apps can be further validated, they offer potential benefits of easier and faster dietary tracking with tools like barcode scanners to log packaged foods [12]. Indeed, recently Wharton et al [25] found greater adherence to dietary tracking with a commercially available diet app (Lose It!) compared with pen and paper.

### Limitations

Our survey was disseminated in English-speaking, developed countries, which may limit the generalizability of our findings. The survey was sent out on different dates to the five participating countries, due to factors outside of our control. For instance, dietetic associations emailed their members at varying frequencies (eg, weekly, fortnightly, or monthly), which influenced when the survey invitation was distributed. Future studies should explore alternative ways of distributing surveys that are independent of administrators - perhaps using social media to disseminate the survey [26,27].

The overall response rate is lower than we desired, however it is similar or greater to electronic surveys of dietitians in participating countries [22,28-30]. The survey distribution period overlapped with the London 2012 Olympic Games, which may have contributed to the low response rate, especially in the UK. Dietitians who use smartphone diet apps may have been more inclined to answer the survey, leading to a possible non-response bias that may have overestimated diet app use [5,31]. However, we made an effort to encourage all potential participants through multiple reminders via email.

We were unable to distinguish whether respondents used diet apps for dietary assessment or dietary tracking. Future surveys should differentiate between these two possible roles of diet apps, as certain apps may be better suited to one of these two tasks. In this study, all of the most commonly used diet apps compare actual nutrient intake to goal recommendations, which may make them prone to social desirability bias as overt nutrition recommendations can encourage people to report that they consume foods inline with known recommendations [32]. Therefore, currently available diet apps may be most suited to *track* dietary intake in order to increase adherence to nutrition recommendations rather than to accurately *assess* dietary intake. Lastly, we did not survey athletes who use diet apps, which would have provided an interesting comparison group. Future studies should investigate the prevalence and perception of diet app use among athletes, especially as a way to record their dietary intake to share with their consulting sports dietitian.

### Conclusions

We found that one-third of sports dietitians used smartphone diet apps with athletes. Sports dietitians who used diet apps had a positive perception of them, and viewed diet apps as useful in helping to assess and track the dietary intake of athletes. We expect that more sports dietitians will use diet apps as smartphone adoption continues to increase and diet app software is refined. Software developers have an opportunity to address the limitations of currently available diet apps and enhance the benefits cited by the sports dietitians in this study. Future research should continue to survey dietitians from all disciplines on their use of smartphone diet apps to examine usage and best practice in dietary assessment and tracking. Finally, there is clearly a need to validate commercially available diet apps, so that dietitians can be confident in recommending their use for dietary assessment and tracking.

## Multimedia Appendix 1

Study questionnaire.

## Abbreviations

PDA: personal digital assistant
