# Supplementary material for: Diet App Use by Sports Dietitians: A Survey in Five Countries
Source: JMIR Mhealth Uhealth. 2015 Jan 22;3(1):e7. doi: 10.2196/mhealth.3345 (PMC4319146; doi:10.2196/mhealth.3345)
Supplement: Supplementary file 1 [file mhealth_v3i1e7_app1.pdf]

Diet Applications

Thank you for your time and consideration. We appreciate your support. Please be assured that your answers are anonymous and treated confidentially.

For each question, click on the circle or square to indicate your chosen answer(s), or type your answer into the text box provided. Click 'NEXT' (bottom of this page) to move to the next page or 'PREVIOUS' to return to the previous page. Questions with an asterix (*) require an answer.

Here is an example of a smartphone diet application (app) that enables users to track dietary intake.

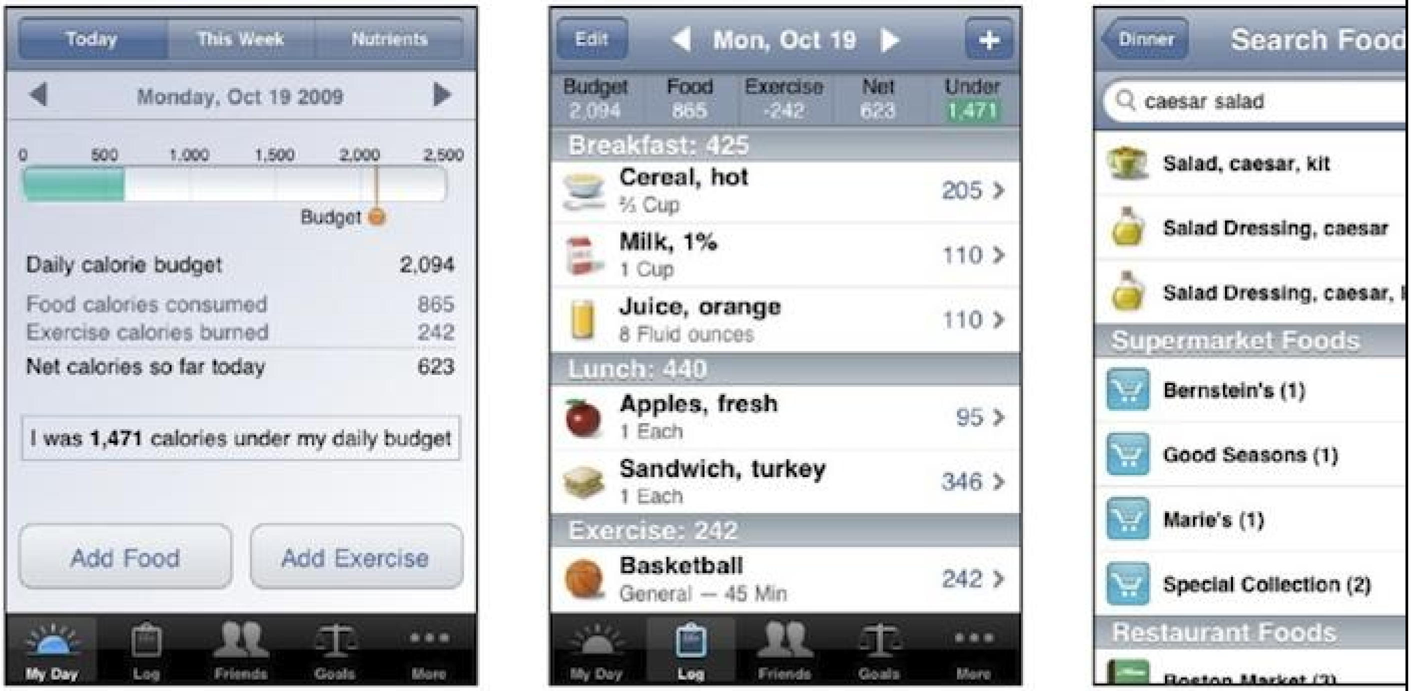

***1. Do you use smartphone diet apps (like the one above) that track dietary intake in your sports nutrition practice?**

- ○ Yes
- ○ No
- ○ Other (please specify)

Diet Apps

For each question, click on the circle or square to indicate your chosen answer(s), or type your answer into the text box provided. Click 'NEXT' (bottom of this page) to move to the next page or 'PREVIOUS' to return to the previous page. Questions with an asterix (*) require an answer.

***2. Which smartphone diet application(s) do you use?**

3. How did you hear about this/these diet app(s)? Please tick all the apply.

- ☐ From a client
- ☐ From a friend
- ☐ Searching the internet
- ☐ From an app store (such as iTunes)
- ☐ From a colleague
- ☐ Other (please specify)

*4. How often do you recommend clients to track their diet using a smartphone diet app?

- ○ Daily
- ○ A few days per week
- ○ A few days per month
- ○ A few days every 2-3 months
- ○ A few days per year
- ○ Certain meals or snacks
- ○ It's up to the client
- ○ Never
- ○ Other (please specify)

*5. Please rate the following:

	Very	Somewhat	Slightly	Not at all	N/A
How **effective** has the smartphone diet app been in assisting **you** to assess the diet of your clients?	○	○	○	○	○
How **effective** has the smartphone diet app been in assisting **your clients** to assess their own diet?	○	○	○	○	○
How **confident** are you in using a smartphone?	○	○	○	○	○

Any comments?

*6. How does the smartphone diet app compare to your traditional (non smartphone) method for the following:

- ○ Smartphone diet app is **better** than traditional method for dietary assessment
- ○ Smartphone diet app is **worse** than traditional method dietary assessment
- ○ Smartphone diet app is **equivalent** to traditional method dietary assessment

7. What are the benefits of using smartphone diet apps?

8. What are the limitations or barriers of using smartphone diet apps?

9. How many clients have you recommended using a smartphone diet app to monitor their dietary intake in the last 3 months?

- 0–5
- 6–10
- 11–15
- 15–20
- More than 20

Any comments?

For each question, click on the circle or square to indicate your chosen answer(s), or type your answer into the text box provided. Click ‘NEXT’ (bottom of this page) to move to the next page or 'PREVIOUS' to return to the previous page. Questions with an asterix (*) require an answer.

*10. Do you assess the dietary intake of your clients?

- Yes
- No

For each question, click on the circle or square to indicate your chosen answer(s), or type your answer into the text box provided. Click ‘NEXT’ (bottom of this page) to move to the next page or 'PREVIOUS' to return to the previous page. Questions with an asterix (*) require an answer.

11. Please indicate whether any of the following are reasons why you do not assess intake

	No	Yes
I have limited access to clients due to their training commitments	○	○
Clients do not return dietary intake information (eg food records)	○	○
I have limited access to food composition databases	○	○
I don't have enough time	○	○
Dietary intake information received from clients is not reliable	○	○
I don't think it is useful to assess dietary intake	○	○
Clients aren't referred for nutrition consultations	○	○

Other (please specify)

Dietary Assessment

For each question, click on the circle or square to indicate your chosen answer(s), or type your answer into the text box provided. Click 'NEXT' (bottom of this page) to move to the next page or 'PREVIOUS' to return to the previous page. Questions with an asterix (*) require an answer.

*12. What method do you most often use to assess dietary intake?

- ○ 24 hour recall
- ○ Diet record
- ○ Diet history (usual diet)
- ○ Food frequency questionnaire
- ○ Other (please specify)

Dietary Assessment

For each question, click on the circle or square to indicate your chosen answer(s), or type your answer into the text box provided. Click 'NEXT' (bottom of this page) to move to the next page or 'PREVIOUS' to return to the previous page. Questions with an asterix (*) require an answer.

13. What tools do your clients use to record their dietary intake? Please tick all that apply.

- ☐ Pen and paper
- ☐ Smartphone or mobile phone
- ☐ Computer - word processing
- ☐ Computer - website
- ☐ Other (please specify)

14. How many days do you usually request your clients to record or recall their dietary intake?

- More than 7 days
- 5–7 days
- 3–4 days
- 2 days
- 1 day
- Other (please specify)

15. How frequently do you usually assess dietary intake in an individual client?

- Yearly
- A few times during the year
- About once a month
- 2-3 times per month
- More than 3 times per month
- Other (please specify)

16. Are any of the following a barrier to assessing dietary intake?

	No	Yes	N/A
Limited access to clients due to their training commitments			
Dietary intake information received from clients is not reliable			
Clients do not return dietary intake information (eg food records)			
Clients aren't referred for nutrition consultations			
I don't have enough time			

Other (please specify)

Converting Food Intake to Nutrient Intake

For each question, click on the circle or square to indicate your chosen answer(s), or type your answer into the text box provided. Click ‘NEXT’ (bottom of this page) to move to the next page or 'PREVIOUS' to return to the previous page. Questions with an asterix (*) require an answer.

*17. With the food intake information that you receive, do you calculate, estimate or neither calculate nor estimate:

	Calculate	Estimate	Neither calculate nor estimate
Current energy intake (for instance kJ or kcal per day) ?	○	○	○
Current macronutrient intake (for instance, grams of carbohydrates per day)?	○	○	○

Any comments?

18. Are any of the following barriers to converting food intake to nutrient intake (for instance, converting lasagna to kJ or kcal of energy)?

	No	Yes
Limited access to food composition databases	○	○
Some foods are not in food composition database	○	○
Clients do not return dietary intake information (eg food records)	○	○
Dietary intake information received from clients is not reliable	○	○
I don't have enough time	○	○

Other (please specify)

Macronutrient Recommendations

For each question, click on the circle or square to indicate your chosen answer(s), or type your answer into the text box provided. Click 'NEXT' (bottom of this page) to move to the next page or 'PREVIOUS' to return to the previous page. Questions with an asterix (*) require an answer.

*19. Do you set targets for energy and/or macronutrient intakes for your clients? For instance, recommended kcal or kJ; grams of carbohydrates, protein and/or fat per day.

○ Yes

○ No

Nutrition Care Process

For each question, click on the circle or square to indicate your chosen answer(s), or type your answer into the text box provided. Click 'NEXT' (bottom of this page) to move to the next page or 'PREVIOUS' to return to the previous page. Questions with an asterix (*) require an answer.

20. Do you consider any of the following factors when setting energy and/or macronutrient targets for your clients?

	No	Yes
American College of Sports Medicine (ACSM) / American Dietetic Association (ADA) Sports Nutrition Position Paper	○	○
Results from dietary assessment	○	○
New Zealand Sports Nutrition Position Paper	○	○
Formulas (such as the Harris-Benedict equation for energy requirements)	○	○
Clients' individual needs (eg sport, position, body composition, medical conditions, etc)	○	○
Personal experience	○	○

Other (please specify)

21. Do you use the American Dietetic Association (ADA) / International Confederation of Dietetic Associations (ICDA) Nutrition Care Process?

- ○ Yes
- ○ No
- ○ I'm not sure

22. Do you use any of the following recommendations or reference standards on a regular basis?

	No	Yes
Clinical Sports Nutrition textbook, edited by Burke & Deakin	○	○
International Society of Sports Nutrition Position Stands	○	○
American College of Sports Medicine (ACSM) / American Dietetic Association (ADA) Sports Nutrition Position Paper	○	○
New Zealand Sports Nutrition Position Paper	○	○
International Olympic Committee (IOC) Consensus Statement	○	○

Other (please specify)

Demographics

For each question, click on the circle or square to indicate your chosen answer(s), or type your answer into the text box provided. Click ‘DONE’ (bottom of this page) once you have completed your questionnaire.

23. How long have you been practicing as a sports dietitian or sports nutritionist?

- Less than 1 year
- 1–5 years
- 6–10 years
- 11–15 years
- 16–20 years
- More than 20 years
- Other (please specify)

24. What are the 3 most common sports that your clients participate in?

Sport 1:

Sport 2:

Sport 3:

25. In what country do you live?

- United Kingdom
- Australia
- United States of America
- Canada
- New Zealand
- Other (please specify)

26. Which category below includes your age?

- 20 or younger
- 21-29
- 30-39
- 40-49
- 50-59
- 60 or older

27. Do you have any final comments?

If you would like to receive a **copy of the results** of this study, as well as a **fact sheet about smartphone diet apps** Click Here

If not, click the **[Done]** button on this end page.
